# Association Between Frailty Index and New-Onset Stroke in Middle-Aged and Elderly Chinese Adults with Gastrointestinal or Liver Diseases: A Longitudinal Cohort Study

**DOI:** 10.3390/jcm15197514

**Published:** 2026-09-27

**Authors:** Xiao She, Haiyan Yang, Xiao Zhao, Longbao Yang

**Affiliations:** 1 Department of Gastroenterology, Xi’an Jiaotong University Second Affiliated Hospital, Xi’an 710004, China; sheandxiao@163.com (X.S.); yhynn010307@163.com (H.Y.); 2 Department of Gastroenterology, First Hospital of Lanzhou University, Lanzhou 730000, China; zhaoxiaodoct@163.com; 3 The First Clinical Medical College, Lanzhou University, Lanzhou 730000, China

**Keywords:** frailty index, stroke, gastrointestinal diseases, liver diseases, cohort study, CHARLS

## Abstract

**Background:** Although the frailty index (FI) has been reported to associate with stroke risk in older adults, its relationship in those with gastrointestinal (GI) or liver diseases remains unclear. We examined this association in a Chinese cohort with these comorbidities. **Methods:** Using baseline data from the China Health and Retirement Longitudinal Study (CHARLS) 2011, we included participants aged ≥45 years with GI or liver diseases. Cox proportional hazards models, restricted cubic splines, and likelihood ratio tests were applied to evaluate the association between FI and new-onset stroke risk. **Results:** A total of 3682 participants were included (mean age 58.78 ± 9.3 years; 55.78% female). Over a mean follow-up of 8 years, 9.26% experienced a stroke. FI was significantly associated with new-onset stroke risk in a non-linear manner (likelihood ratio test *p* < 0.001), with an exploratory threshold identified at FI = 0.18 (95% CI: 0.1–0.2). Below this threshold, each 0.1-unit increment in FI was strongly associated with elevated stroke risk (HR = 2.10, 95% CI: 1.56–2.82, *p* < 0.0001); above it, the association plateaued and was no longer significant (HR = 1.10, 95% CI: 0.99–1.22, *p* = 0.079). These results remained robust across subgroup and sensitivity analyses. **Conclusions:** This study demonstrates a significant non-linear association between FI and new-onset stroke risk in middle-aged and elderly individuals with GI or liver diseases, highlighting the importance of FI assessment in this population.

## 1. Introduction

According to the Global Burden of Disease Study 2019, there were approximately 12.2 million new stroke cases globally in 2019, making stroke the second leading cause of death, accounting for 11.6% of total deaths. Although age-standardized incidence and mortality rates for stroke decreased from 1990 to 2019, mortality rates and disability-adjusted life years (DALYs) in low-income countries remain significantly higher [1]. Specifically, the World Stroke Organization report for 2025 indicates a significant increase in the global burden of stroke from 1990 to 2021, with 87.0% of stroke-related deaths and 89.0% of DALYs occurring in low-income and lower-middle-income countries [2]. In China, there were 3.94 million new stroke cases in 2019, an increase of 86.0% in incidence since 1990, while the age-standardized incidence rate decreased by 9.3% [3]. Research indicates that the burden of stroke among individuals aged 55 and older is gradually increasing due to low physical activity levels and high body mass index, especially in the context of global population growth and aging, highlighting the urgency for attention to this issue [4].

The frailty index (FI) is an important indicator of overall health status, reflecting an individual’s vulnerability to stressors through the assessment of declines across multiple physiological, psychological, and functional domains [5,6]. Wang et al. found that in several international cohorts focusing on the elderly population, the FI demonstrated significant changes both before and after stroke, particularly an increase during the occurrence of stroke events, underscoring the importance of FI in assessing high stroke risk in older adults [7]. Additionally, other studies have shown that in the Chinese elderly population, both baseline frailty index and clustering analyses based on long-term frailty index trajectories are significantly correlated with stroke risk, highlighting the importance of considering FI in stroke risk assessments [8]. In nationally representative cohorts from the United States and China, it was observed that there is a nonlinear relationship between the frailty index and stroke risk in the elderly population; however, the lack of a clearly defined threshold may impact the FI’s utility as a risk assessment tool in clinical practice [9].

The relationship between gastrointestinal (GI) diseases and stroke risk has attracted growing attention. Roth et al. found that GI diseases are significantly associated with the risk of first-time ischemic stroke, particularly functional, inflammatory, and infectious diseases of the stomach [10]. Regarding liver disease, the association between cirrhosis and stroke risk has been a subject of considerable interest. A systematic review and meta-analysis demonstrated that patients with cirrhosis have a significantly elevated risk of hemorrhagic stroke [11]. Furthermore, cirrhosis has been identified as an independent risk factor for in-hospital mortality in patients with ischemic stroke [12]. These findings underscore the importance of proactive cirrhosis management in stroke prevention and care. Separately, Che et al. reported that higher FI scores are associated with an increased risk of non-neoplastic digestive system diseases, highlighting the clinical relevance of FI assessment in elderly patients with comorbid GI conditions [13]. Given the interplay among FI, GI diseases, and stroke risk, investigating this association in middle-aged and elderly patients with GI or liver diseases is therefore warranted.

The China Health and Retirement Longitudinal Study (CHARLS) is a nationally representative cohort study encompassing middle-aged and elderly individuals in China. The CHARLS provides comprehensive data on health status, socioeconomic factors, and long-term follow-up [14], making it well suited for examining the frailty–stroke relationship. Although existing CHARLS-based research has established an association between FI and new-onset stroke risk [8,9], studies focusing specifically on individuals with GI or liver diseases remain scarce. To address this gap, the present study analyzed CHARLS participants with GI or liver diseases at baseline to examine the association between FI and subsequent stroke risk.

## 2. Methods

### 2.1. Study Design and Population

This study employed a prospective cohort design, drawing data from the CHARLS, which encompasses five time points conducted in 2011, 2013, 2015, 2018, and 2020. The baseline survey, conducted from June 2011 to March 2012, included approximately 17,707 individuals from 10,000 households across 150 counties [14]. We focused on analyzing data from this baseline wave, selecting participants who self-reported having GI or liver diseases. Following Chen et al. [15], GI and liver diseases are a series of common diseases occurring in the digestive system that share several risk factors, including poor dietary habits, poor health behaviors, and environmental factors. While excluding individuals with a history of stroke, new cases of stroke were identified through follow-up assessments conducted in subsequent waves. The CHARLS (http://charls.pku.edu.cn/, accessed on 1 December 2025) received ethical approval from the Institutional Review Board of Peking University Health Science Center (IRB00001052-11015), and all participants provided written informed consent [14].

The study employed the following exclusion criteria: first, participants with non-GI diseases or liver diseases were excluded, totaling 13,560 individuals; second, 147 individuals aged under 45 years were excluded; third, 35 participants with a history of stroke at baseline were excluded; finally, 283 individuals lacking at least one follow-up assessment were excluded. Ultimately, a total of 3682 participants were included in the analysis (Figure 1). Furthermore, this study adhered to the Strengthening the Reporting of Observational Studies in Epidemiology (STROBE) guidelines for cohort study reporting.

### 2.2. Frailty Index Assessment

This study employed the FI, a standardized age-associated deficit accumulation model [6], to assess frailty status, which is based on 32 items related to self-reported health conditions, functional limitations, and biomarkers. These items include physician-diagnosed health issues (such as hypertension, diabetes, and cancer), daily living difficulties (such as dressing, bathing, and eating), and mobility challenges (such as walking and climbing stairs). (See Table S1 for a complete list of all 32 deficit items.) Additionally, the 10-item Center for Epidemiological Studies-Depression (CES-D10) questionnaire was used to evaluate depressive symptoms, along with memory and orientation tests to assess cognitive function. In the coding scheme, 0 indicates no deficit, while 1 indicates the presence of a deficit. The cognitive score was transformed into a continuous scale ranging from 0 to 1, with lower values indicating better functional status. Each participant’s FI was calculated by dividing the total number of health deficits by 32, with scores ranging from 0 to 1 [13]. Based on previous research, participants were categorized into Robust (FI ≤ 0.10), Pre-frail (0.10 < FI < 0.25), and Frail (FI ≥ 0.25) groups [13,16,17]. Although participants with prevalent stroke were excluded from the analytic sample, the stroke item was retained in the FI to maintain the integrity of the standardized 32-item structure. Since all participants were free of stroke at baseline, this item was universally scored as 0. Such uniform scoring corresponds to a linear scaling (FI_31_ = FI_32_ × 32/31) that preserves relative ranking of participants and does not affect threshold identification, hazard ratio (HR) estimation (which depends on within-unit FI increments rather than absolute scale), or clinical classification. Therefore, retaining this item does not materially affect the main findings.

### 2.3. Incidence of New-Onset Stroke

New-onset stroke was defined as self-reported physician-diagnosed events during the follow-up periods (2013, 2015, 2018, and 2020). During each follow-up, trained staff posed standardized questions to participants to confirm the occurrence of stroke [18,19], specifically: (i) Have you been told by your doctor that you have been diagnosed with a stroke? (ii) When was the condition first diagnosed/known to you? (iii) Are you currently receiving any follow-up treatment for your stroke? Participants who reported experiencing a stroke event were classified as stroke patients, and the time of their self-reported onset was recorded as years or age. Time-to-event was derived from each participant’s self-reported onset date (i.e., the response to survey question ii above), rather than from the wave at which stroke was first ascertained. In cases where a participant reported a stroke at multiple follow-up waves with inconsistent onset dates, the earliest self-reported onset date was used as the reference time of event. If no stroke event occurred, the date of last follow-up was recorded as the censoring time. Time-to-event was calculated as the interval between this reference date and the baseline examination date.

### 2.4. GI and Liver Diseases Measurements

In this study, the assessment of GI and liver diseases was based on health status and functional questionnaires from the baseline survey. The questionnaire included the questions: “Have you been diagnosed with digestive disease (tumor or cancer not included) by a doctor?” and “Have you been diagnosed with liver disease (fatty liver, cancer, and tumor not included) by a doctor?” Participants who answered “yes” were defined as having the respective diseases. This assessment method is consistent with standards established in the previous literature [15].

### 2.5. Covariates

Based on previous literature [9,13] and clinical relevance, the covariates in this study were derived from data collected during the baseline survey of the CHARLS database, encompassing the participants’ demographic characteristics and health status. The covariates included: age (in years), sex (female and male), body mass index (BMI, calculated as weight in kilograms divided by the square of height in meters, kg/m^2^), residential area (urban and rural), education level (illiterate, primary, secondary, and college or above), marital status (without spouse and with spouse), alcohol consumption (never, current, and former drinkers), smoking status (never, current, and former smokers), and annual income (in RMB, categorized into three groups: <15,000 yuan, 15,000–25,000 yuan, and ≥25,000 yuan).

The specific definitions of chronic diseases are as follows: hypertension is defined as a systolic blood pressure ≥ 140 mmHg or diastolic blood pressure ≥ 90 mmHg or currently receiving antihypertensive treatment [20]. Diabetes mellitus is diagnosed based on self-reports from physicians, with fasting plasma glucose ≥ 126 mg/dL, HbA1c ≥ 6.5%, or currently taking glucose-lowering medications. Meeting any of these criteria is sufficient to diagnose diabetes [21]. Dyslipidemia is characterized by elevated triglycerides (≥150 mg/dL), total cholesterol (≥240 mg/dL), low high-density lipoprotein cholesterol (<40 mg/dL), or high low-density lipoprotein cholesterol (≥160 mg/dL). It also includes individuals who are currently on lipid-lowering medications and those diagnosed with dyslipidemia by a healthcare professional [21]. Depressive symptoms are assessed using the CES-D10 Scale, with a score ≥ 10 defined as the presence of depressive symptoms [22].

In addition, the study collected laboratory biomarkers, including: serum glucose (GLU, mg/dL), hemoglobin (HGB, g/dL), total cholesterol (TC, mg/dL), triglycerides (TG, mg/dL), high-density lipoprotein cholesterol (HDL-C, mg/dL), low-density lipoprotein cholesterol (LDL-C, mg/dL), C-reactive protein (CRP, mg/dL), and estimated glomerular filtration rate (eGFR, mL/min/1.73 m^2^), to further assess the health status of participants.

### 2.6. Statistical Analysis

Continuous variables were presented as means ± standard deviations (SD) or medians (interquartile ranges), while categorical variables were reported as frequencies and percentages. The FI was categorized into three groups based on established thresholds: Robust (FI ≤ 0.10), Pre-frail (FI between 0.11 and 0.25), and Frail (FI ≥ 0.25). The statistical significance of group differences was assessed using one-way analysis of variance (ANOVA) for normally distributed continuous variables or the Kruskal–Wallis H test for non-normally distributed continuous variables; categorical variables were compared using the chi-square test.

Univariable and multivariable analyses were conducted using Cox proportional hazards models to evaluate the associations between baseline characteristics and the risk of new-onset stroke, calculating HR and 95% confidence intervals (CI) for each variable. Based on existing literature [9,13] and clinical relevance, a multivariable Cox model was used to further explore potential influencing factors, adjusting for variables including age, sex, residential area, alcohol consumption status, smoking status, annual income category, BMI, hypertension, diabetes, dyslipidemia, and several biochemical indicators (e.g., serum glucose, CRP, hemoglobin, TC, TG, LDL-C, and eGFR).

For missing data, if any covariate had missing values exceeding 2%, dummy variables were created to handle these missing data [18,23,24]. For categorical variables, an additional category was created to represent missing data; for continuous variables with substantial missing values, a two-variable approach was implemented: one variable that included the original values with missing values replaced by zero, and another indicator variable to identify which observations had missing data.

To investigate the non-linear relationship between FI and the risk of new-onset stroke, we applied restricted cubic splines based on the Cox model for smooth curve fitting, controlling for confounding factors. A piecewise linear regression model was used to examine the threshold effect of FI on stroke risk. Additionally, likelihood ratio tests were performed to compare the performance of the univariable linear regression and the piecewise linear regression models [25,26], complemented by bootstrap resampling methods to assess the robustness of the identified inflection points [27], thereby enhancing the validity of the study’s results.

Adjusted cumulative hazard curves for new-onset stroke across different frailty status groups were estimated using the fully adjusted Cox proportional hazards model. Additionally, the Cox regression model evaluated the association between FI and the risk of new-onset stroke, incorporating stratified analyses based on participant characteristics. Participants were grouped according to the following features: age (<65 years and ≥65 years), sex (male and female), BMI (underweight, normal, overweight, and obese), residential area (urban and rural), education level (illiterate, middle school, vocational school, college and above), marital status (with spouse and without spouse), alcohol consumption habits (never, current, and past), smoking habits (never, current, and past), annual income (<15,000 RMB, 15,000–25,000 RMB, and ≥25,000 RMB), and comorbidities including hypertension, diabetes, dyslipidemia, depression, as well as liver and GI diseases. The proportional hazards assumption for the Cox model was assessed using Schoenfeld residuals. The global test showed no significant violation (χ^2^ = 22.30, df = 25, *p* = 0.619), confirming that the proportional hazards assumption was met.

Sensitivity analyses were conducted to assess the robustness of the study findings. First, to assess the potential impact of overadjustment arising from the inclusion of hypertension and diabetes as both FI components and model covariates, a sensitivity analysis was conducted repeating the primary Cox regression with these two variables excluded from covariate adjustment. Second, participants with follow-up times of less than two years were excluded, resulting in a final analysis cohort of 3472 patients. Third, an alternative method for handling missing data—multiple imputation using a random forest-based approach—was employed as a sensitivity analysis focusing on the threshold effect analysis. The imputation was performed incorporating all covariates included in the fully adjusted model, generating five imputed datasets, each comprising the full analysis cohort of 3682 participants. The threshold effect analysis was performed separately on each of the five imputed datasets, and the resulting estimates were pooled using Rubin’s rules to account for between-imputation uncertainty. Fourth, to account for the potential influence of competing risks, a Fine & Gray subdistribution hazard model was applied with death treated as the competing event.

All statistical analyses were performed using EmpowerStats (www.empowerstats.com, accessed on 10 December 2025; X&Y Solutions, Inc., Boston, MA, USA) and R software version 4.2.0 (http://www.r-project.org). A two-sided significance level of *p* < 0.05 was considered statistically significant.

## 3. Results

### 3.1. Characteristics of Study Population

This study included a total of 3682 middle-aged and elderly participants, with an average age of 58.78 ± 9.13 years, of whom 55.78% were female, and the average BMI was 23.04 ± 3.72 kg/m^2^ (Table 1). Of these participants, 3154 (85.66%) had GI disease only, 315 (8.56%) had liver disease only, and 213 (5.78%) had both conditions. Based on the frailty index, participants were classified into three groups: Robust group (*N* = 977), Pre-frail group (*N* = 1683), and Frail group (*N* = 1022). The results showed that, compared with the Robust and Pre-frail groups, the Frail group had a significantly older average age (*p* < 0.001), a higher proportion of rural residents (*p* < 0.001), and a lower education level (66.83% illiteracy, *p* < 0.001). In terms of health status, the Frail group exhibited significantly higher rates of hypertension (39.64%), diabetes (11.69%), dyslipidemia (15.52%), and depression (81.28%) compared with the Robust and Pre-frail groups (all *p* < 0.001). Additionally, compared with the Pre-frail and Robust groups, the Frail group showed significantly elevated levels of GLU, TC, TG, LDL-C, and CRP (all *p* < 0.05), while the eGFR was significantly lower (*p* < 0.001).

### 3.2. The Cumulative Incidence of New-Onset Stroke

During a follow-up period of approximately 8.0 years (7.95 ± 2.05 years), a total of 341 participants reported new-onset stroke, resulting in a cumulative incidence of 9.26% (Table 1). Among different frailty status groups, the cumulative incidence of new-onset stroke was as follows: 4.40% (43 cases) in the Robust group, 9.21% (155 cases) in the Pre-frail group, and 13.99% (143 cases) in the Frail group. The differences in the cumulative incidence of new-onset stroke among these groups were statistically significant (*p* < 0.001).

### 3.3. Univariate and Multivariate Analysis

According to the univariate analysis, the risk of new-onset stroke was associated with the following factors: age, BMI, education level, marital status, hypertension, diabetes, depression, FI, HGB, TC, TG, LDL-C, CRP, and eGFR (all *p* < 0.05) (Table S2).

In the fully adjusted Model 3 (Table 2), the baseline FI was significantly associated with the risk of new-onset stroke. For each 0.1 increase in the FI, the HR was 1.24 (95% CI: 1.14–1.33, *p* < 0.0001). Compared with Robust individuals, Pre-frail individuals had a HR of 1.93 (95% CI: 1.35–2.75, *p* = 0.0003). The HR for Frail individuals was 2.70 (95% CI: 1.85–3.95, *p* < 0.0001). The trend for increasing risk with worsening frailty status was statistically significant (*p* for trend < 0.0001).

### 3.4. Non-Linear Relationship Between FI and the Risk of New-Onset Stroke

Figure 2 illustrates the nonlinear relationship between the FI and the risk of new-onset stroke. In the initial phase, an increase in FI is associated with a significant rise in the risk of new-onset stroke, indicating that at lower levels of FI, the increase in risk is substantial. However, as FI continues to rise, the rate of increase in risk gradually diminishes, ultimately stabilizing. Notably, the HR below the exploratory inflection point (HR = 1881.57) represents a statistical artifact due to mathematical amplification and should be interpreted with caution; a per 0.1-unit increase in FI below the threshold corresponds to a more statistically robust HR of 2.10 (95% CI: 1.56–2.82).

Using a two-piecewise linear regression model, we determined that the exploratory inflection point for FI in relation to new-onset stroke risk is 0.18 (95% CI: 0.1–0.2, likelihood ratio test *p* < 0.001) among the 3606 participants with complete covariate data included in the fully adjusted analysis. On the left side of the inflection point (FI < 0.18), each 0.1-unit increase in FI was significantly associated with an increased risk of new-onset stroke (HR = 2.10, 95% CI: 1.56–2.82, *p* < 0.0001). On the right side of the inflection point (FI ≥ 0.18), the association attenuated and was no longer statistically significant (HR = 1.10, 95% CI: 0.99–1.22, *p* = 0.079) (Table 3). Given that this exploratory inflection point was identified empirically within the current dataset, further external validation is warranted before it can be applied as a clinical reference threshold.

### 3.5. Adjusted Cumulative Hazard of New-Onset Stroke by Frailty Status

The adjusted cumulative hazard curves, derived from a fully adjusted Cox proportional hazards model, showed that participants in the Frail group had a higher cumulative hazard of new-onset stroke compared with those in the Pre-frail and Robust groups (Figure S1). A clear stepwise gradient was observed across frailty categories, with individuals in the Frail group demonstrating a steeper increase in cumulative hazard over the study period, indicating that greater frailty was associated with both higher overall stroke risk and faster risk accumulation.

### 3.6. Subgroup Analysis

Exploratory subgroup analyses suggested that the positive association between frailty status and new-onset stroke risk was broadly consistent across subgroups defined by age, sex, area of residence, education level, marital status, drinking and smoking status, income, BMI, hypertension, dyslipidemia, depression, and disease type (GI or liver diseases), with Frail individuals consistently showing higher HRs compared with Pre-frail individuals relative to the Robust group (Table S3). These analyses were unadjusted and are intended to be hypothesis-generating; apparent differences in effect estimates across subgroups should be interpreted with caution, as no formal interaction tests were performed.

### 3.7. Sensitivity Analysis

The sensitivity analysis was consistent with the main analysis. Firstly, to address the potential concern of overadjustment arising from the inclusion of hypertension and diabetes as both FI components and model covariates, we conducted a sensitivity analysis repeating the primary Cox regression with these two variables excluded from covariate adjustment. The results remained highly consistent with the primary analysis (Table S4), confirming that overadjustment did not materially influence our findings. Secondly, we excluded individuals with a follow-up time of less than two years, involving a total of 3472 participants. We adjusted for factors such as age, sex, area of residence, drinking status, smoking status, annual income categories, BMI, hypertension, diabetes, dyslipidemia, and various biochemical markers. This analysis confirmed a non-linear relationship between the FI and the risk of new-onset stroke (Table S5). Thirdly, we employed multiple imputation methods to address missing covariate values, generating five imputed datasets, each comprising the full analysis cohort of 3682 participants. The threshold effect analysis was performed on each imputed dataset separately, and the estimates were pooled using Rubin’s rules (Table S6). The inflection point (K) was identical across all five imputed datasets (K = 0.18), and the pooled estimates were consistent with those obtained in the primary analysis, further supporting the robustness of our findings. Fourthly, to account for the potential influence of competing risks, we repeated the primary analysis using a competing risk regression model with death as the competing event. The results remained consistent with the main analysis (Table S7), suggesting that the observed associations between FI, frailty status, and new-onset stroke risk were not materially affected by competing mortality.

## 4. Discussion

The present study demonstrates that frailty, as quantified by a 32-item frailty index, is independently associated with new-onset stroke risk in older Chinese adults with GI or liver diseases, following a nonlinear dose–response pattern with more pronounced effects at lower frailty levels. Stratified analyses by frailty status further revealed that, relative to Robust individuals, the HR for stroke was 2.20 (95% CI: 1.57–3.08, *p* < 0.0001) in the Pre-frail group and 3.70 (95% CI: 2.63–5.20, *p* < 0.0001) in the Frail group. These findings are consistent with the hypothesis that the accumulation of health deficits captured by the FI heightens stroke susceptibility through multi-system dysfunction, and align with prior longitudinal evidence linking frailty to adverse outcomes including hospitalization, disability, and mortality [28,29,30].

Multiple studies support the close association between frailty status and the risk of cardiovascular events, including stroke. In a study involving 154,696 participants, Farooqi et al. found that the incidence of cardiovascular events (including new or recurrent myocardial infarction, stroke, and heart failure) and all-cause mortality was significantly higher in the Frail group compared with the Non-frail group, indicating that frailty may be an independent risk factor for cardiovascular events [31]. Additionally, research has shown that frailty is commonly present among hypertensive patients and is closely linked to various health issues, such as cardiovascular diseases [32]. Furthermore, in patients with atrial fibrillation, frailty has been associated with an increased risk of stroke. Gugganig et al. noted that the adjusted HR for stroke risk in the Frail group was 3.29, and the study also found significant associations between frailty and the risks of unplanned hospitalization and all-cause mortality [33].

In this context, a recent study by Zhang et al. [9] further provided evidence of a significant association between the FI and the risk of stroke in individuals aged 45 and older. Specifically, for every 0.1-unit increase in FI, the odds of new-onset stroke increased by 2.90 times (95% CI: 2.35–3.57) in the NHANES cohort, and by 1.78 times (95% CI: 1.63–1.85) in the CHARLS cohort [9]. Our study yielded similar findings in the elderly population in China, showing that for every 0.1-unit increase in FI, the HR for new-onset stroke was 1.24 (95% CI: 1.14–1.33). Notably, our study focused on individuals with concomitant GI or liver diseases in the baseline survey, providing unique insights that extend beyond the general elderly population. In subgroup analyses with different characteristics, the Frail group exhibited the highest stroke risk, illustrating a stable relationship between frailty status and the risk of new-onset stroke. Consistent with Zhang et al.’s conclusion of a non-linear relationship between FI and the occurrence of stroke [9], we further explored the segmented effects and found that, in the elderly population with GI or liver diseases, the exploratory inflection point for FI and new-onset stroke was 0.18 (95% CI: 0.1–0.2).

The observed nonlinear association indicates that when the FI is below 0.18, each 0.1-unit increment in FI is associated with a HR of 2.10 (95% CI: 1.56–2.82, *p* < 0.0001) for stroke risk. In contrast, when the FI exceeds this exploratory inflection threshold, the HR for stroke risk is 1.10 (95% CI: 0.99–1.22, *p* = 0.079). These findings indicate that the impact of frailty on cerebrovascular health exhibits a threshold effect: even modest increases in frailty at lower FI levels are associated with markedly elevated stroke risk, underscoring the particular vulnerability of this subgroup. This may be attributed to the fact that, in the context of underlying digestive system or liver diseases, frailty can lead to a marked decline in physiological function, rendering these patients more susceptible to stroke events. However, beyond this threshold, additional worsening of frailty may not significantly elevate stroke risk, possibly due to the influence of other pathophysiological mechanisms and the complex interplay of multiple comorbidities affecting the health status of elderly patients.

The FI is a multidimensional tool that comprehensively captures an individual’s physiological and biochemical vulnerability. How FI interacts with GI or liver diseases to influence stroke risk warrants further investigation. One potential mechanism involves the gut microbiota: GI diseases may alter microbial composition, which in turn may elevate stroke risk—a relationship that has garnered increasing attention in the recent literature. Studies have reported that gut microbiota can influence the host’s metabolic state and produce neuroactive compounds, which modulate neural function and, through bidirectional communication along the gut–brain axis, affect the pathogenesis of ischemic stroke [34]. Furthermore, GI diseases may impact the integrity of the gut barrier, subsequently affecting stroke risk. Damage to the gut barrier can lead to the translocation of gut microbiota, triggering systemic immune responses and inhibiting recovery from stroke [35]. In elderly populations, frailty is closely associated with changes in gut microbiota composition [36]. The gut microbiota of Frail individuals plays a crucial role in regulating low-grade chronic inflammation [37]. Thus, changes in gut microbiota may serve as an important bridge connecting frailty and stroke risk in patients with GI diseases. Although this study did not directly design analyses related to gut microbiota, existing research indicates that modulating gut microbiota is considered a potential strategy for the prevention and treatment of stroke [38,39]. For instance, restructuring gut microecology through pharmacological combinations can improve neurological function after stroke and alleviate related complications, further supporting the significance of gut health in stroke recovery [40].

Declines in frailty and muscle mass may be closely related to poor prognoses in patients with liver disease through their impact on liver metabolic capacity [41]. As the level of FI rises, increased inflammatory markers and the deterioration of vascular health may exacerbate endothelial dysfunction, thereby heightening the risk of atherosclerosis, which may lead to cerebrovascular events [42,43,44,45]. Additionally, FI is closely related to daily functional capacity in the elderly [46]; declines in functional capacity can restrict patients’ activity levels, increasing stroke risk [47]. Lastly, FI is not only a physiological indicator but also encompasses an individual’s psychological and social adaptability. Psychosocial factors, such as depression and anxiety, may also be associated with the risk of cardiovascular and cerebrovascular diseases [48,49]. This study confirms that in patients with comorbid depression, the risk of new-onset stroke gradually increases with the rising degree of frailty. Notably, the biological basis of the FI has been validated through Mendelian randomization studies, which demonstrate that genetically predicted FI is associated with an increased risk of any type of stroke (OR = 1.360, 95% CI: 1.006–1.838, *p* = 0.046), particularly showing a significant relationship with the risk of large-artery atherosclerotic stroke [50]. It is worth noting that in patients with GI or liver diseases, frailty does not act in isolation; rather, it synergizes with the underlying pathological milieu of these conditions—including chronic systemic inflammation, impaired nutritional absorption, and metabolic dysregulation—to amplify cerebrovascular risk. This biological convergence may explain why the association between FI and stroke risk was observed to be particularly notable in this organ-specific population; however, as this study did not include a direct comparison group without GI or liver diseases, this interpretation remains speculative and requires confirmation in future comparative studies.

This study underscores the importance of frailty assessment in identifying populations at varying risk levels, particularly among older adults with GI or liver diseases. The identification of a non-linear association with an exploratory threshold enhances our understanding of frailty’s role in stroke pathogenesis in the context of organ-specific vulnerabilities. While these findings suggest potential value in incorporating frailty assessment within clinical monitoring frameworks, the application of this exploratory threshold to clinical practice requires validation in external cohorts before it can be adopted as a clinical reference point. Prospective interventional studies are needed to evaluate whether frailty-targeted strategies may impact stroke risk reduction in this population. Future research should prioritize external validation of these findings and dynamic monitoring of frailty trajectories in this population, given evidence linking longitudinal FI changes to stroke risk in older adults [8].

### Study Limitations

Despite the strengths of this study, several important limitations must be acknowledged.

Recall bias and misclassification of disease status. This study relied on self-reported diagnoses of GI or liver diseases without objective diagnostic verification, and stroke outcomes were similarly self-reported. While both misclassification and recall bias are potential concerns, non-differential misclassification typically biases associations toward the null, attenuating rather than exaggerating the observed associations. Consequently, any significant associations detected likely represent conservative estimates of the true effect. Furthermore, sensitivity analyses conducted over the extended follow-up period yielded consistent results, thereby helping to mitigate concerns regarding recall bias.

Extreme estimates in threshold analysis and scale extrapolation. The per-unit HR below the exploratory inflection point (HR = 1881.57, 95% CI: 92.71–38,186.67) represents a statistical artifact rather than a genuine biological effect, attributable to mathematical amplification rather than data sparsity. Since the frailty index is bounded between 0 and 1, and participants below the inflection point were confined to a narrow FI range (0–0.18), extrapolating the segment-specific slope to a full one-unit increment results in substantial inflation of the HR. In contrast, the exploratory inflection point at FI = 0.18 provides an empirical demarcation within our study population, indicating the threshold below which stroke risk increases more steeply, and above which the association attenuates and loses statistical significance. Therefore, the per 0.1-unit hazard ratio (HR = 2.10, 95% CI: 1.56–2.82) represents a more appropriate and statistically robust effect size for the segment below this threshold; however, its application to clinical practice requires external validation in independent cohorts.

Potential for unmeasured confounding. Although we adjusted for major potential confounders—including age, sex, residential area, alcohol consumption, smoking status, annual income, body mass index, and prevalent diagnoses of hypertension, diabetes, and hyperlipidemia—as well as several biochemical indicators, unmeasured or incompletely measured confounders may still influence the observed associations. These include detailed medication history, additional chronic conditions, and other lifestyle or environmental exposures.

Missing data in covariates. Some covariates exhibited missing data, which was addressed using dummy variable coding for the primary analysis and multiple imputation for sensitivity analyses. While these methods were employed to minimize bias, they remain subject to assumptions regarding the missing data mechanism.

Population specificity and generalizability. This study is limited to a Chinese population, and the relationship between frailty and stroke may differ across diverse racial and ethnic groups due to genetic, environmental, or socioeconomic differences. Future multi-center studies involving diverse populations could address this limitation and provide a more comprehensive understanding of the frailty-stroke association. Additionally, future research should explore potential mediating and moderating factors—such as medication use, additional lifestyle factors, and psychosocial variables—to further elucidate the underlying mechanisms.

Despite these limitations, the consistent findings across sensitivity analyses and the use of validated measures strengthen our confidence in the main findings regarding the non-linear relationship between frailty and stroke risk.

## 5. Conclusions

This study demonstrated a significant non-linear association between the frailty index and new-onset stroke risk among middle-aged and elderly adults with GI or liver diseases. An exploratory threshold effect was identified, below which frailty was strongly and significantly associated with stroke risk, while above this threshold the association plateaued and was no longer statistically significant. These findings highlight the importance of frailty assessment in this population.

## Figures and Tables

**Figure 1:**
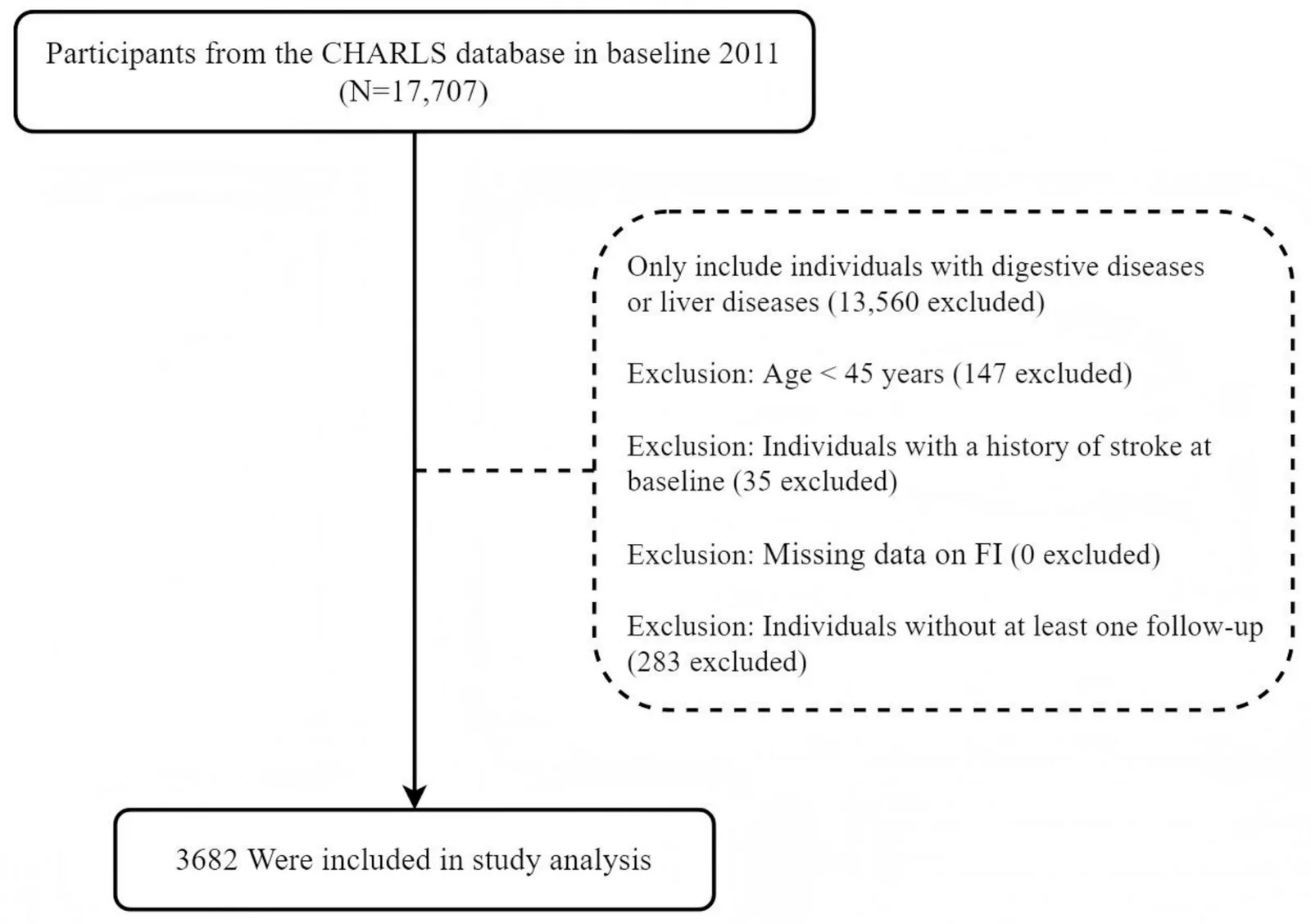
Flowchart of participant selection. CHARLS, China Health and Retirement Longitudinal Study; FI, frailty index.

**Figure 2:**
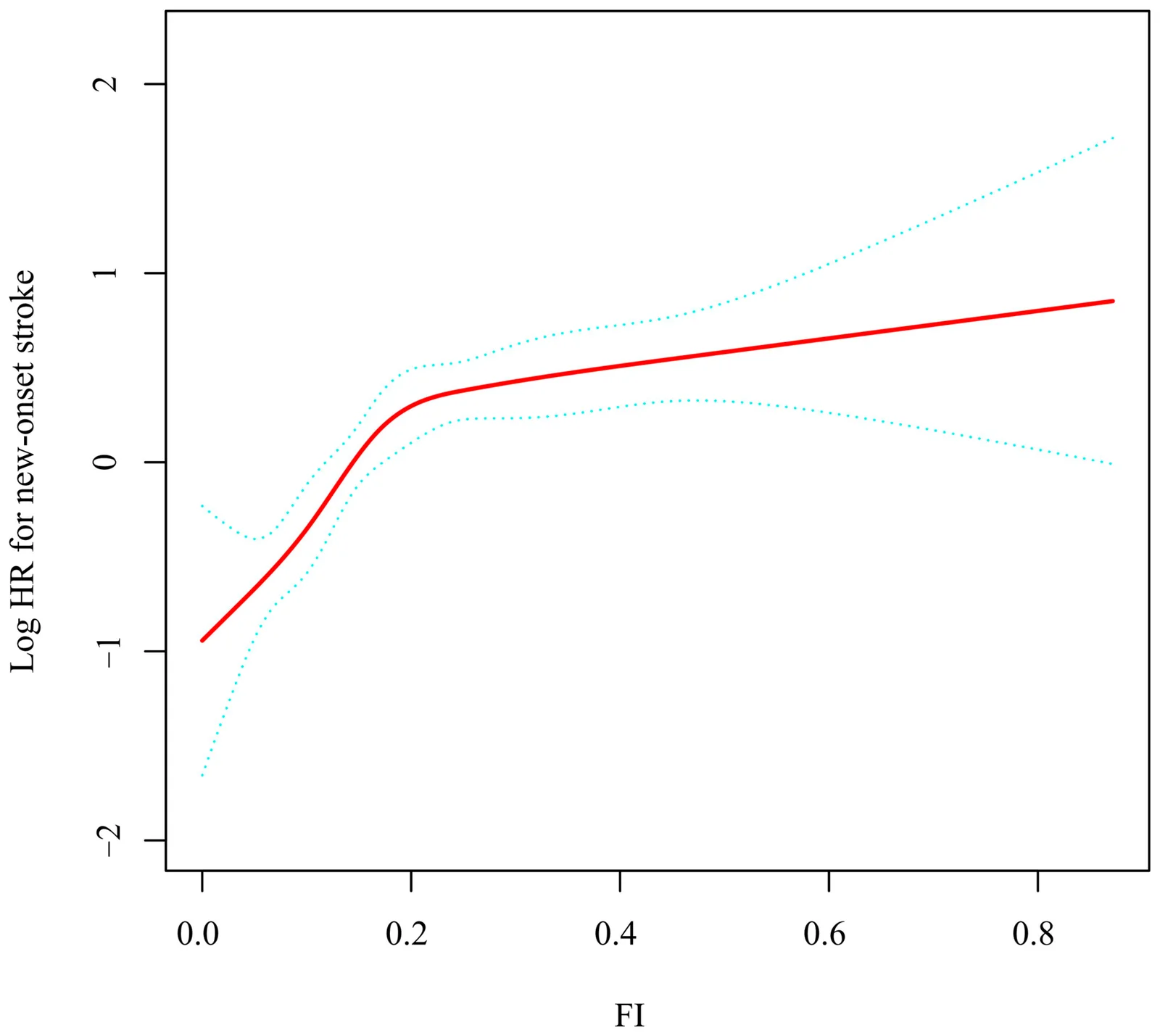
Restricted cubic spline analysis illustrating the association between FI and new-onset stroke risk in patients with GI or liver diseases. The solid red line represents the smooth curve fit between variables, while the dotted blue lines denote the 95% CI. Adjusted for age (years), sex, area of residence, drinking status, smoking status, annual income categories, BMI, hypertension, diabetes, dyslipidemia, and various biochemical markers (Glu, CRP, HGB, TC, TG, LDL, eGFR). HR, hazard ratio; FI, frailty index; GI, gastrointestinal; CI, confidence interval; BMI, body mass index; Glu, glucose levels; CRP, C-reactive protein; HGB, hemoglobin; TC, total cholesterol; TG, triglycerides; LDL, low-density lipoprotein; eGFR, estimated glomerular filtration rate.

**Table 1:** Baseline characteristics of study participants stratified by frailty status.

Variables	Overall *N* = 3682	Frailty Status			
		Robust (≤0.10) *N* = 977	Pre-Frail (0.11 to 0.25) *N* = 1683	Frail (≥0.25) *N* = 1022	*p*-Value
Age (years)	58.78 ± 9.13	55.47 ± 7.94	58.37 ± 8.49	62.63 ± 9.80	<0.001
Sex					<0.001
Female	2054 (55.78%)	421 (43.09%)	952 (56.57%)	681 (66.63%)	
Male	1628 (44.22%)	556 (56.91%)	731 (43.43%)	341 (33.37%)	
BMI (kg/m^2^)	23.04 ± 3.72	22.98 ± 3.67	23.00 ± 3.66	23.15 ± 3.85	0.587
Area of residence					<0.001
Urban	1310 (35.58%)	454 (46.47%)	590 (35.06%)	266 (26.03%)	
Rural	2372 (64.42%)	523 (53.53%)	1093 (64.94%)	756 (73.97%)	
Education					<0.001
Illiterate	1759 (47.81%)	274 (28.07%)	802 (47.71%)	683 (66.83%)	
Primary	822 (22.34%)	234 (23.98%)	386 (22.96%)	202 (19.77%)	
Middle	699 (19.00%)	282 (28.89%)	319 (18.98%)	98 (9.59%)	
College or higher	399 (10.85%)	186 (19.06%)	174 (10.35%)	39 (3.82%)	
Marital status					<0.001
No spouse	407 (11.05%)	45 (4.61%)	166 (9.86%)	196 (19.18%)	
Married with spouse	3275 (88.95%)	932 (95.39%)	1517 (90.14%)	826 (80.82%)	
Drinking					<0.001
Never	2226 (60.57%)	548 (56.21%)	1017 (60.54%)	661 (64.80%)	
Current	1094 (29.77%)	353 (36.21%)	515 (30.65%)	226 (22.16%)	
Ever	355 (9.66%)	74 (7.59%)	148 (8.81%)	133 (13.04%)	
Smoking					<0.001
Never	2257 (63.13%)	539 (59.82%)	1039 (62.63%)	679 (66.90%)	
Current	1010 (28.25%)	287 (31.85%)	487 (29.36%)	236 (23.25%)	
Ever	308 (8.62%)	75 (8.32%)	133 (8.02%)	100 (9.85%)	
Annual income groups (in yuan)					<0.001
<15,000	1302 (35.36%)	194 (19.86%)	619 (36.78%)	489 (47.85%)	
15,000–25,000	283 (7.69%)	65 (6.65%)	124 (7.37%)	94 (9.20%)	
≥25,000	695 (18.88%)	227 (23.23%)	310 (18.42%)	158 (15.46%)	
Hypertension					<0.001
No	2725 (74.58%)	869 (89.50%)	1247 (74.49%)	609 (60.36%)	
Yes	929 (25.42%)	102 (10.50%)	427 (25.51%)	400 (39.64%)	
Diabetes					<0.001
No	3400 (93.48%)	948 (98.04%)	1561 (93.98%)	891 (88.31%)	
Yes	237 (6.52%)	19 (1.96%)	100 (6.02%)	118 (11.69%)	
Dyslipidemia					<0.001
No	3134 (87.10%)	878 (91.36%)	1423 (86.19%)	833 (84.48%)	
Yes	464 (12.90%)	83 (8.64%)	228 (13.81%)	153 (15.52%)	
Depression					<0.001
No	1724 (50.17%)	695 (84.55%)	844 (51.91%)	185 (18.72%)	
Yes	1712 (49.83%)	127 (15.45%)	782 (48.09%)	803 (81.28%)	
Digestive disease					0.154
No	315 (8.56%)	94 (9.62%)	147 (8.73%)	74 (7.24%)	
Yes	3367 (91.44%)	883 (90.38%)	1536 (91.27%)	948 (92.76%)	
Liver disease					0.212
No	3125 (85.55%)	842 (86.45%)	1438 (86.00%)	845 (83.91%)	
Yes	528 (14.45%)	132 (13.55%)	234 (14.00%)	162 (16.09%)	
GLU (mg/dL)	108.20 ± 32.45	105.99 ± 29.37	106.67 ± 28.84	112.70 ± 39.55	<0.001
HGB (g/dL)	14.31 ± 2.18	14.54 ± 2.17	14.31 ± 2.16	14.13 ± 2.19	0.003
TC (mg/dL)	192.66 ± 38.95	188.89 ± 38.91	192.94 ± 37.74	195.45 ± 40.75	0.008
TG (mg/dL)	103.54 (74.34–154.88)	96.46 (69.69–159.30)	105.32 (75.22–149.57)	107.08 (78.76–159.30)	0.015
HDL-C (mg/dL)	51.75 ± 15.26	51.21 ± 15.15	51.58 ± 14.75	52.50 ± 16.17	0.260
LDL-C (mg/dL)	115.77 ± 34.74	112.55 ± 33.68	117.48 ± 34.25	115.67 ± 36.29	0.015
CRP (mg/dL)	0.93 (0.51–1.99)	0.85 (0.46–1.72)	0.93 (0.52–1.97)	0.98 (0.54–2.50)	<0.001
eGFR (mL/min/1.73 m^2^)	92.73 ± 14.61	94.82 ± 13.62	93.27 ± 14.14	90.02 ± 15.78	<0.001
Follow-up time (Year)	7.95 ± 2.05	8.28 ± 1.76	8.03 ± 1.97	7.51 ± 2.34	<0.001
New-onset stroke					<0.001
No	3341 (90.74%)	934 (95.60%)	1528 (90.79%)	879 (86.01%)	
Yes	341 (9.26%)	43 (4.40%)	155 (9.21%)	143 (13.99%)	

Data are presented as *n* (%) for categorical variables and mean ± SD or median (interquartile range) for continuous variables. Among the 3682 participants, the amount of missing values for the covariates were 694 (18.86%) for BMI, 3 (0.08%) for education, 7 (0.19%) for drinking status, 107 (2.90%) for smoking status, 1402 (38.07%) for annual income groups, 28 (0.76%) for hypertension, 45 (1.22%) for diabetes, 84 (2.28%) for dyslipidemia, 246 (6.26%) for depressive symptoms, 29 (0.78%) for liver disease, 1091 (29.62%) for GLU, 1109 (30.10%) for HGB, 1085 (29.48%) for TC, 1085 (29.48%) for TG, 1084 (29.43%) for HDL-C, 1087 (29.50%) for LDL-C, 1084 (29.43%) for CRP, and 1095 (29.67%) for eGFR. BMI: body mass index; GLU: serum glucose; HGB: hemoglobin; TC: total cholesterol; TG: triglycerides; HDL-C: high-density lipoprotein cholesterol; LDL-C: low-density lipoprotein cholesterol; CRP: C-reactive protein; eGFR: estimated glomerular filtration rate; SD: standard deviation.

**Table 2:** Association between baseline FI and frailty status with the risk of new-onset stroke.

Variables	Model 1		Model 2		Model 3	
	HR (95%CI)	*p*-Value	HR (95%CI)	*p*-Value	HR (95%CI)	*p*-Value
FI per 0.1	1.33 (1.25, 1.42)	<0.0001	1.26 (1.17, 1.35)	<0.0001	1.24 (1.14, 1.33)	<0.0001
Frailty status						
Robust	Reference		Reference		Reference	
Pre-frail	2.20 (1.57, 3.08)	<0.0001	2.06 (1.45, 2.90)	<0.0001	1.93 (1.35, 2.75)	0.0003
Frail	3.70 (2.63, 5.20)	<0.0001	3.00 (2.08, 4.32)	<0.0001	2.70 (1.85, 3.95)	<0.0001
*P* for trend		<0.0001		<0.0001		<0.0001

Model 1 was adjusted for: None; Model 2 was adjusted for age (years), sex, area of residence, drinking status, smoking status, annual income categories, and BMI; and Model 3 was adjusted for hypertension, diabetes, dyslipidemia, and various biochemical markers (glucose, C-reactive protein, hemoglobin, total cholesterol, triglycerides, low-density lipoprotein, estimated glomerular filtration rate) based on Model 2. FI, frailty index; HR, hazard ratio; CI, confidence interval; BMI, body mass index; Glu, glucose levels; CRP, C-reactive protein; HGB, hemoglobin; TC, total cholesterol; TG, triglycerides; LDL, low-density lipoprotein; eGFR, estimated glomerular filtration rate.

**Table 3:** Threshold effect analysis of FI and risk of new-onset stroke (total *N* = 3606).

Models	Per-Unit Increase		Per 0.1-Unit Increase	
	HR (95%CI)	*p*-Value	HR (95%CI)	*p*-Value
Model I				
One line effect	8.32 (3.86, 17.94)	<0.0001	1.24 (1.14, 1.33)	<0.0001
Model II				
Turning point (K)	0.18		1.83	
<K	1881.57 (92.71, 38,186.67)	<0.0001	2.10 (1.56, 2.82)	<0.0001
≥K	2.59 (0.93, 7.20)	0.0688	1.10 (0.99, 1.22)	0.0793
*P* for likelihood ratio test *		<0.001		<0.001
95% CI for turning point	0.1–0.2		1.0–2.0	

Data are presented as HR (95% CI) and *p*-value; Model I, linear analysis; Model II, non-linear analysis. Adjusted for age (years), sex, area of residence, drinking status, smoking status, annual income categories, BMI, hypertension, diabetes, dyslipidemia, and various biochemical markers (Glu, CRP, HGB, TC, TG, LDL, eGFR). * *p* < 0.05 indicates that Model II is significantly different from Model I. Restricted cubic spline smoothing was applied. In total, participants with missing values greater than 2% were treated using dummy variable coding. These variables included BMI, annual income, smoking status, fasting blood glucose, C-reactive protein, hemoglobin, TC, TG, LDL-C, and eGFR. Covariates with missing values less than 2% included drinking status, hypertension, and diabetes, which collectively resulted in the exclusion of 76 participants. The final sample size for the threshold effect analysis was 3606 participants. The per-unit HR below the inflection point (HR = 1881.57, 95% CI: 92.71–38,186.67) should be interpreted with caution, as it represents a statistical artifact arising from data sparsity at the lower tail of the FI distribution, where the piecewise model extrapolates across the full unit range despite observed values clustering within a much narrower interval; the per 0.1-unit HR (2.10, 95% CI: 1.56–2.82) is the more robust estimate for this segment. FI, frailty index; HR, hazard ratio; CI, confidence interval; BMI, body mass index; Glu, glucose levels; CRP, C-reactive protein; HGB, hemoglobin; TC, total cholesterol; TG, triglycerides; LDL, low-density lipoprotein; eGFR, estimated glomerular filtration rate.

## Data Availability

Publicly available datasets were analyzed in this study. The data are available from the China Health and Retirement Longitudinal Study (CHARLS) at http://charls.pku.edu.cn/ (accessed on 1 December 2025).

## Supplementary Materials

The following supporting information can be downloaded at https://www.mdpi.com/article/10.3390/jcm15197514/s1: Figure S1. Cumulative hazard of new-onset stroke (%) by FI categories adjusted for confounding factors. Table S1. The 32 items used to construct the frailty index. Table S2. Univariate analysis of baseline variables associated with new-onset stroke. Table S3. Association of frailty status with new-onset stroke risk in different subgroups. Table S4. Sensitivity analysis of the association between baseline frailty index and frailty status with the risk of new-onset stroke (Excluding hypertension and diabetes from covariate adjustment to avoid overadjustment with FI components). Table S5. Threshold effect analysis—sensitivity analysis (Excluding participants with follow-up time less than 2 years, fully adjusted, *N* = 3472). Table S6. Threshold effect analysis with multiple imputation (fully adjusted, *N* = 3682). Table S7. Association between baseline FI and frailty status with the risk of new-onset stroke using competing risk regression (death as the competing event).

## Abbreviations

The following abbreviations are used in this manuscript:

ANOVA	Analysis of Variance
BMI	Body Mass Index
CHARLS	China Health and Retirement Longitudinal Study
CI	Confidence Interval
CRP	C-reactive Protein
CES-D10	The 10-item Center for Epidemiological Studies-Depression
DALYs	Disability-Adjusted Life Years
eGFR	Estimated Glomerular Filtration Rate
FI	Frailty Index
GI	Gastrointestinal
GLU	Serum Glucose
HGB	Hemoglobin
HR	Hazard Ratio
LDL-C	Low-Density Lipoprotein Cholesterol
TC	Total Cholesterol
TG	Triglycerides
HDL-C	High-Density Lipoprotein Cholesterol
