# Supplementary material for: Association Between Frailty Index and New-Onset Stroke in Middle-Aged and Elderly Chinese Adults with Gastrointestinal or Liver Diseases: A Longitudinal Cohort Study
Source: J Clin Med. 2026 Sep 27;15(19):7514. doi: 10.3390/jcm15197514 (PMC13665714; doi:10.3390/jcm15197514)
Supplement: Supplementary file 1 [file jcm-15-07514-s001.zip › jcm-4550009-supplementary.pdf]

Supplementary Materials

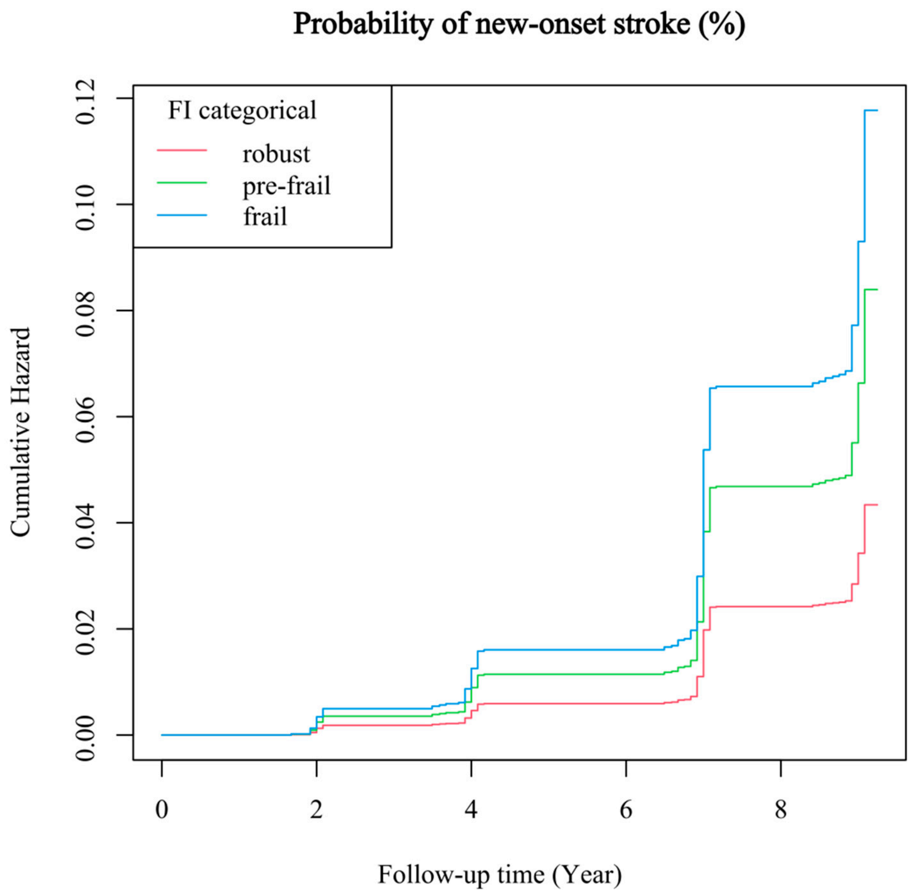

Figure S1. Cumulative hazard of new-onset stroke (%) by FI categories adjusted for confounding factors.

Adjusted for age (years), sex, area of residence, drinking status, smoking status, annual income categories, BMI, hypertension, diabetes, dyslipidemia, and various biochemical markers (Glu, CRP, HGB, TC, TG, LDL, eGFR). FI, frailty index; BMI, body mass index; Glu, glucose levels; CRP, C-reactive protein; HGB, hemoglobin; TC, total cholesterol; TG, triglycerides; LDL, low-density lipoprotein; eGFR, estimated glomerular filtration rate.

Table S1. The 32 items used to construct the frailty index.

Number	Description of the item	Cut-off value
1	Self-reported physician diagnosed hypertension	Yes = 1, No = 0
2	Self-reported physician diagnosed diabetes	Yes = 1, No = 0
3	Self-reported physician diagnosed cancer	Yes = 1, No = 0
4	Self-reported physician diagnosed arthritis	Yes = 1, No = 0
5	Self-reported physician diagnosed lung disease	Yes = 1, No = 0
6	Self-reported physician diagnosed asthma	Yes = 1, No = 0
7	Self-reported physician diagnosed memory-related disease	Yes = 1, No = 0
8	Self-reported physician diagnosed heart problem	Yes = 1, No = 0
9	Self-reported physician diagnosed stroke	Yes = 1, No = 0
10	Self-reported physician diagnosed psych problem	Yes = 1, No = 0
11	Self-reported hearing problems	Yes = 1, No = 0
12	Self-reported vision problems	Yes = 1, No = 0
13	Self-reported general health status	Poor/Fair = 1, Good/Very good = 0
14	Difficulty with dressing	Yes = 1, No = 0
15	Difficulty with bathing or showering	Yes = 1, No = 0
16	Difficulty with eating	Yes = 1, No = 0
17	Difficulty with getting in and out of bed	Yes = 1, No = 0
18	Difficulty with using the toilet	Yes = 1, No = 0
19	Difficulty with managing money	Yes = 1, No = 0
20	Difficulty with taking medication	Yes = 1, No = 0
21	Difficulty with shopping for groceries	Yes = 1, No = 0
22	Difficulty with cleaning house	Yes = 1, No = 0
23	Difficulty with preparing meals	Yes = 1, No = 0
24	Mobility: difficulty with walking 100 yards or one block	Yes = 1, No = 0
25	Mobility: difficulty with getting up from a chair after sitting for long periods	Yes = 1, No = 0
26	Mobility: difficulty with climbing several flights of stairs without resting	Yes = 1, No = 0
27	Mobility: difficulty with lifting or carrying weights over 10 pounds/jins	Yes = 1, No = 0
28	Mobility: difficulty with picking up a coin from the table	Yes = 1, No = 0
29	Mobility: difficulty with stooping, kneeling, or crouching	Yes = 1, No = 0

30	Mobility: difficulty with reaching arms above shoulder level	Yes = 1, No = 0
31	Depression: CESD-10 questionnaire	CESD-10 >10 = 1, ≤10 = 0
32	Cognition: (memory test score + orientation test score) / 14	Continuous variable, ranging from 0 to 1

Depression was assessed using the 10-item Center for Epidemiologic Studies Depression Scale (CESD-10) in the China Health and Retirement Longitudinal Study (CHARLS), with total scores ranging from 0 to 30.

The memory score (range: 0-10) was calculated as the average number of words not recalled across immediate and delayed recall tasks. The orientation test assessed temporal awareness through four items (day of week, month, date, and year), with one point awarded for each incorrect response (range: 0-4).

Frailty Index Calculation: The frailty index (FI) was calculated by dividing the total number of health deficits (sum of all 32 items) by 32, with scores ranging from 0 to 1. Higher values indicate greater frailty.

Table S2. Univariate analysis of baseline variables associated with new-onset stroke.

**Variables**	**Statistics**	**HR (95%CI)**	***P*-value**
Age (years)	58.78 ± 9.13	1.04 (1.03, 1.05)	<0.0001
Sex			
Female	2054 (55.78%)	Reference	
Male	1628 (44.22%)	0.91 (0.73, 1.13)	0.3870
BMI (kg/m^2)	23.04 ± 3.72	1.06 (1.03, 1.08)	0.0001
Area of residence			
Urban	1310 (35.58%)	Reference	
Rural	2372 (64.42%)	0.93 (0.75, 1.17)	0.5506
Education			
Illiterate	1759 (47.81%)	Reference	
Primary	822 (22.34%)	0.94 (0.72, 1.22)	0.6503
Middle	699 (19.00%)	0.63 (0.45, 0.86)	0.0039
College or higher	399 (10.85%)	0.76 (0.52, 1.11)	0.1556
Marital status			
No spouse	407 (11.05%)	Reference	
Married with spouse	3275 (88.95%)	0.65 (0.48, 0.88)	0.0058
Drinking			
Never	2226 (60.57%)	Reference	
Current	1094 (29.77%)	0.85 (0.67, 1.09)	0.1979
Ever	355 (9.66%)	1.03 (0.72, 1.48)	0.8701
Smoking			
Never	2257 (63.13%)	Reference	
Current	1010 (28.25%)	1.01 (0.79, 1.29)	0.9406
Ever	308 (8.62%)	1.52 (1.08, 2.14)	0.0169
Annual income groups (in yuan)			
< 15000	1302 (35.36%)	Reference	
15000-25000	283 (7.69%)	0.87 (0.57, 1.31)	0.4937
≥ 25000	695 (18.88%)	0.97 (0.73, 1.29)	0.8422
Hypertension			
No	2725 (74.58%)	Reference	
Yes	929 (25.42%)	2.11 (1.70, 2.63)	<0.0001
Diabetes			
No	3400 (93.48%)	Reference	
Yes	237 (6.52%)	1.88 (1.33, 2.66)	0.0003
Dyslipidemia			
No	3134 (87.10%)	Reference	

Yes	464 (12.90%)	1.69 (1.29, 2.22)	0.0001
Depression			
No	1724 (50.17%)	Reference	
Yes	1712 (49.83%)	1.53 (1.22, 1.91)	0.0002
Liver disease			
No	3125 (85.55%)	Reference	
Yes	528 (14.45%)	1.21 (0.91, 1.62)	0.1871
Digestive disease			
No	315 (8.56%)	Reference	
Yes	3367 (91.44%)	0.89 (0.62, 1.29)	0.5362
GLU (mg/dL)	108.20 ± 32.45	1.00 (1.00, 1.01)	0.1025
HGB (g/dL)	14.31 ± 2.18	1.06 (1.01, 1.12)	0.0288
TC (mg/dL)	192.66 ± 38.95	1.01 (1.00, 1.01)	0.0010
TG (mg/dL)	131.83 ± 103.32	1.00 (1.00, 1.00)	0.0160
HDL-C (mg/dL)	51.75 ± 15.26	0.99 (0.98, 1.00)	0.1163
LDL-C (mg/dL)	115.77 ± 34.74	1.01 (1.00, 1.01)	0.0019
CRP (mg/dL)	2.32 ± 5.43	1.02 (1.01, 1.04)	0.0029
eGFR (mL/min/1.73 m²)	92.73 ± 14.61	0.98 (0.98, 0.99)	0.0001
FI	0.19 ± 0.14	17.85 (9.42, 33.83)	<0.0001
Frailty status			
Robust	977 (26.53%)	Reference	
Pre-frail	1683 (45.71%)	2.20 (1.57, 3.08)	<0.0001
Frail	1022 (27.76%)	3.70 (2.63, 5.20)	<0.0001

Data are expressed as the mean ± SD, or percentage. HR, hazard ratio; CI, confidence interval; BMI, body mass index; HGB, hemoglobin; TC, total cholesterol; TG, triglycerides; HDL-C, high-density lipoprotein cholesterol; LDL-C, low-density lipoprotein cholesterol; CRP, C-reactive protein; eGFR, estimated glomerular filtration rate; FI, frailty index. *P* values less than 0.05 are considered statistically significant.

Table S3. Association of frailty status with new-onset stroke risk in different subgroups.

Characteristic	N	Robust	Pre-frail		Frail	
			HR (95%CI)	*P*-value	HR (95%CI)	*P*-value
Age (years)						
< 65	2777	Reference	2.28 (1.54, 3.38)	<0.0001	4.14 (2.76, 6.19)	<0.0001
≥ 65	905	Reference	1.67 (0.86, 3.24)	0.1293	2.03 (1.06, 3.89)	0.0323
Sex						
Female	2054	Reference	2.25 (1.31, 3.85)	0.0031	4.56 (2.69, 7.71)	<0.0001
Male	1628	Reference	2.31 (1.49, 3.58)	0.0002	2.86 (1.74, 4.68)	<0.0001
BMI (kg/m^2)						
Underweight	261	Reference	1.63 (0.18, 14.58)	0.6630	9.09 (1.17, 70.49)	0.0347
Normal weight	1917	Reference	1.94 (1.22, 3.09)	0.0051	2.57 (1.58, 4.19)	0.0002
Overweight	704	Reference	1.94 (0.95, 3.99)	0.0707	3.19 (1.54, 6.60)	0.0018
Obese	106	Reference	1.44 (0.36, 5.81)	0.6065	2.11 (0.54, 8.20)	0.2806
Area of residence						
Urban	1310	Reference	2.39 (1.44, 3.98)	<0.0007	4.23 (2.48, 7.22)	<0.0001
Rural	2372	Reference	2.14 (1.36, 3.37)	0.0011	3.62 (2.30, 5.70)	<0.0001
Education						
Illiterate	1759	Reference	1.65 (0.98, 2.79)	0.0605	2.17 (1.29, 3.65)	0.0037
Primary	822	Reference	3.48 (1.55, 7.78)	0.0025	7.56 (3.37, 16.95)	<0.0001
Middle	699	Reference	2.58 (1.20, 5.53)	0.0148	5.38 (2.33, 12.43)	<0.0001
College or higher	399	Reference	1.43 (0.63, 3.26)	0.3944	5.24 (2.13, 12.93)	0.0003
Marital status						
No spouse	407	Reference	1.48 (0.56, 3.91)	0.4243	1.09 (0.41, 2.92)	0.8671
Married with spouse	3275	Reference	2.21 (1.54, 3.17)	<0.0001	4.18 (2.91, 6.01)	<0.0001
Drinking						
Never	2226	Reference	2.65 (1.66, 4.25)	<0.0001	4.31 (2.69, 6.91)	<0.0001
Current	1094	Reference	1.58 (0.93, 2.69)	0.0884	2.48 (1.39, 4.42)	0.0021
Ever	355	Reference	3.29 (0.74, 14.57)	0.1171	6.17 (1.44, 26.50)	0.0144
Smoking						
Never	2257	Reference	2.10 (1.33, 3.32)	0.0015	3.64 (2.31, 5.75)	<0.0001
Current	1010	Reference	1.65 (0.95, 2.88)	0.0774	2.43 (1.33, 4.42)	0.0037
Ever	308	Reference	3.56 (1.04, 12.15)	0.0427	6.10 (1.81, 20.63)	0.0036
Annual income groups (in yuan)						
< 15000	1302	Reference	2.14 (1.10, 4.16)	0.0255	3.16 (1.62, 6.13)	0.0007
15000-25000	283	Reference	9.16 (1.21, 69.62)	0.0323	10.10 (1.30, 78.28)	0.0269

≥ 25000	695	Reference	1.94 (0.99, 3.79)	0.0523	4.19 (2.14, 8.18)	<0.0001
Hypertension						
No	2725	Reference	1.89 (1.28, 2.77)	0.0012	3.30 (2.22, 4.92)	<0.0001
Yes	929	Reference	2.28 (1.04, 4.99)	0.0387	3.05 (1.40, 6.64)	0.0050
Diabetes						
No	3400	Reference	2.10 (1.49, 2.96)	<0.0001	3.54 (2.49, 5.03)	<0.0001
Yes	237	Reference	[a]	0.9970	[a]	0.9969
Dyslipidemia						
No	3134	Reference	2.12 (1.47, 3.05)	<0.0001	3.53 (2.44, 5.11)	<0.0001
Yes	464	Reference	2.40 (0.93, 6.18)	0.0702	3.71 (1.44, 9.57)	0.0066
Depression						
No	1724	Reference	2.14 (1.41, 3.25)	0.0004	3.41 (2.00, 5.81)	<0.0001
Yes	1712	Reference	1.35 (0.67, 2.70)	0.3995	2.39 (1.21, 4.71)	0.0118
Liver diseases						
No	3125	Reference	2.27 (1.57, 3.28)	<0.0001	3.76 (2.59, 5.46)	<0.0001
Yes	528	Reference	1.87 (0.80, 4.37)	0.1497	3.38 (1.47, 7.78)	0.0043
Digestive diseases						
No	315	Reference	1.91 (0.69, 5.29)	0.2154	3.63 (1.28, 10.32)	0.0155
Yes	3367	Reference	2.24 (1.57, 3.21)	<0.0001	3.73 (2.60, 5.35)	<0.0001

All HR presented are unadjusted values from exploratory analyses intended to be hypothesis-generating rather than confirmatory. Apparent differences in effect estimates across subgroups should be interpreted with caution, as no formal interaction tests were performed and some subgroups had limited sample sizes resulting in wide confidence intervals. [a] The model failed because of the small sample size. HR, hazard ratio; CI, confidence interval; BMI, body mass index.

Table S4. Sensitivity analysis of the association between baseline frailty index and frailty status with the risk of new-onset stroke (Excluding hypertension and diabetes from covariate adjustment to avoid overadjustment with FI components).

**Variables**	**Model 1**		**Model 2**		**Model 3**	
	**HR (95%CI)**	***P*-value**	**HR (95%CI)**	***P*-value**	**HR (95%CI)**	***P*-value**
FI per 0.1	1.33 (1.25, 1.42)	<0.0001	1.26 (1.17, 1.35)	<0.0001	1.26 (1.17, 1.36)	<0.0001
Frailty status						
Robust	Reference		Reference		Reference	
Pre-frail	2.20 (1.57, 3.08)	<0.0001	2.06 (1.45, 2.90)	<0.0001	2.00 (1.41, 2.82)	<0.0001
Frail	3.70 (2.63, 5.20)	<0.0001	3.00 (2.08, 4.32)	<0.0001	2.96 (2.05, 4.27)	<0.0001
P for trend		<0.0001		<0.0001		<0.0001

Model 1 was adjusted for: None; Model 2 was adjusted for age (years), sex, area of residence, drinking status, smoking status, annual income categories, and BMI; and Model 3 was adjusted for dyslipidemia, and various biochemical markers (glucose, C-reactive protein, hemoglobin, total cholesterol, triglycerides, low-density lipoprotein, estimated glomerular filtration rate) based on Model 2. FI, frailty index; HR, hazard ratio; CI, confidence interval; BMI, body mass index; Glu, glucose levels; CRP, C-reactive protein; HGB, hemoglobin; TC, total cholesterol; TG, triglycerides; LDL, low-density lipoprotein; eGFR, estimated glomerular filtration rate.

Table S5. Threshold effect analysis – sensitivity analysis (Excluding participants with follow-up time less than 2 years, fully adjusted, $N = 3472$).

Models	Per-unit increase		Per 0.1-unit increase	
	HR (95%CI)	*P*-value	HR (95%CI)	*P*-value
Model I				
One line effect	7.56 (3.42, 16.72)	<0.0001	1.22 (1.13, 1.33)	<0.0001
Model II				
Turning point (K)	0.19		1.85	
< K	1079.11 (62.65, 18588.37)	<0.0001	2.06 (1.53, 2.76)	<0.0001
≥ K	2.15 (0.72, 6.40)	0.1680	1.08 (0.97, 1.21)	0.1427
P for likelihood ratio test*		<0.001		<0.001

Data are presented as HR (95% CI) and p-value; Model I represents linear analysis, while Model II indicates non-linear analysis. Adjusted for age (years), sex, area of residence, drinking status, smoking status, annual income categories, BMI, hypertension, diabetes, dyslipidemia, and various biochemical markers (Glu, CRP, HGB, TC, TG, LDL, eGFR). *$P < 0.05$ indicates a significant difference between Model II and Model I. Restricted cubic spline smoothing was applied. FI, frailty index; HR, hazard ratio; CI, confidence interval; BMI, body mass index; Glu, glucose levels; CRP, C-reactive protein; HGB, hemoglobin; TC, total cholesterol; TG, triglycerides; LDL, low-density lipoprotein; eGFR, estimated glomerular filtration rate.

Table S6. Threshold effect analysis with multiple imputation (fully adjusted, N = 3682).

Models	Per-unit increase		Per 0.1-unit increase	
	**HR (95%CI)**	***P*-value**	**HR (95%CI)**	***P*-value**
Model I				
One line effect	8.66 (4.06, 18.46)	<0.0001	1.24 (1.15, 1.34)	<0.0001
Model II				
Turning point (K)	0.18		1.84	
< K	1832.41 (93.82, 35788.08)	<0.0001	2.09 (1.56, 2.79)	<0.0001
≥ K	2.70 (0.98, 7.41)	0.0538	1.10 (0.99, 1.22)	0.0710
P for likelihood ratio test*		<0.001		<0.001

Data are presented as HR (95% CI) and p-value; Model I represents linear analysis, while Model II indicates non-linear analysis. Adjusted for age (years), sex, area of residence, drinking status, smoking status, annual income categories, BMI, hypertension, diabetes, dyslipidemia, and various biochemical markers (Glu, CRP, HGB, TC, TG, LDL, eGFR). *P < 0.05 indicates a significant difference between Model II and Model I. Restricted cubic spline smoothing was applied. Five imputed datasets were generated using a random forest-based multiple imputation approach; estimates from the linear and threshold effect models were pooled across all five datasets according to Rubin's rules. The inflection point (K) was identical across all five imputed datasets (K = 0.18), indicating a highly stable threshold estimate. FI, frailty index; HR, hazard ratio; CI, confidence interval; BMI, body mass index; Glu, glucose levels; CRP, C-reactive protein; HGB, hemoglobin; TC, total cholesterol; TG, triglycerides; LDL, low-density lipoprotein; eGFR, estimated glomerular filtration rate.

Table S7. Association between baseline FI and frailty status with the risk of new-onset stroke using competing risk regression (death as the competing event).

**Variables**	**Model 3**	
	**HR (95%CI)**	***P*-value**
FI per 0.1	1.18 (1.07, 1.30)	0.0006
Frailty status		
Robust	Reference	
Pre-frail	2.39 (1.57, 3.62)	<0.0001
Frail	3.16 (2.01, 4.97)	<0.0001
P for trend		<0.0001

Model 3 was adjusted for age (years), sex, area of residence, drinking status, smoking status, annual income categories, BMI, hypertension, diabetes and dyslipidemia. FI, frailty index; HR, hazard ratio; CI, confidence interval; BMI, body mass index.
